# IMMIGRATION, RESILIENCE, AND ORAL-HEALTH-RELATED QUALITY OF LIFE AMONG CHINESE AMERICAN OLDER ADULTS IN HAWAII

**DOI:** 10.1093/geroni/igz038.2188

**Published:** 2019-11-08

**Authors:** Bei Wu, Yaolin Pei, Wei Zhang

**Affiliations:** 1 New York University, New York, New York, United States; 2 University of Hawaii at Manoa, Honolulu, Hawaii, United States

## Abstract

Very few studies have compared oral health status between the US-born and foreign-born immigrant older adults. Using data collected among 430 Chinese older adults age 55+ residing in Hawai’i, we examined the association between immigrant status and oral health related quality of life (OHQoL) and the moderating role of resilience in linking the association. Controlling for some key covariates, our study results show that US-born Chinese immigrant older adults had better OHQoL than their foreign born counterparts. Factors such as higher level of education (graduate degree or higher), better self-reported health status and no significant tooth loss were related to better OHQoL. The association between immigrant status and OHQoL was moderated by resilience. Specially, resilience was positively and significantly associated with OHQoL among U.S.-born older adults but not among the foreign-born ones. Our findings indicate the importance of immigration and resilience in shaping oral health outcomes among older Chinese Americans.

